# Models of asthma: density-equalizing mapping and output benchmarking

**DOI:** 10.1186/1745-6673-3-S1-S7

**Published:** 2008-02-27

**Authors:** Julia-Annik Börger, Niko Neye, Cristian Scutaru, Carolin Kreiter, Clemens Puk, Tanja C Fischer, Beatrix Groneberg-Kloft

**Affiliations:** 1 Institute of Occupational Medicine, Charité-Universitätsmedizin Berlin, Free University Berlin and Humboldt-University Berlin, Ostpreussendamm 111, D-12207 Berlin, Germany; 2 Department of Dermatology and Allergy, Charité-Universitätsmedizin Berlin, Free University Berlin and Humboldt-University Berlin, Charitéplatz 1, D-10117 Berlin, Germany; 3 Otto-Heubner-Centre, Charité-Universitätsmedizin Berlin, Free University Berlin and Humboldt-University Berlin, Augustenburger Platz 1 OR 1, D-13353 Berlin, Germany

## Abstract

Despite the large amount of experimental studies already conducted on bronchial asthma, further insights into the molecular basics of the disease are required to establish new therapeutic approaches. As a basis for this research different animal models of asthma have been developed in the past years. However, precise bibliometric data on the use of different models do not exist so far. Therefore the present study was conducted to establish a data base of the existing experimental approaches. Density-equalizing algorithms were used and data was retrieved from a Thomson Institute for Scientific Information database. During the period from 1900 to 2006 a number of 3489 filed items were connected to animal models of asthma, the first being published in the year 1968. The studies were published by 52 countries with the US, Japan and the UK being the most productive suppliers, participating in 55.8% of all published items. Analyzing the average citation per item as an indicator for research quality Switzerland ranked first (30.54/item) and New Zealand ranked second for countries with more than 10 published studies. The 10 most productive journals included 4 with a main focus allergy and immunology and 4 with a main focus on the respiratory system. Two journals focussed on pharmacology or pharmacy. In all assigned subject categories examined for a relation to animal models of asthma, immunology ranked first. Assessing numbers of published items in relation to animal species it was found that mice were the preferred species followed by guinea pigs. In summary it can be concluded from density-equalizing calculations that the use of animal models of asthma is restricted to a relatively small number of countries. There are also differences in the use of species. These differences are based on variations in the research focus as assessed by subject category analysis.

## Introduction

Allergic asthma is a complex inflammatory condition of the lung with an increasing prevalence and incidence. Amongst other lung diseases [1-7] the disorder constitutes a major occupational burden of disease [8]. The disease is often concomitant with other allergic diseases such as allergic rhinitis, atopic dermatitis and allergic eye diseases. The direct medical costs evolved from allergic airway inflammation are increasing over the past decades and constitute about an estimated 1-3% of the health fund of the U.S. The economic burden amounts to roughly 12 billion dollar [9-11]. Despite the large amount of experimental studies already conducted on allergic asthma, further insights into the molecular basics of the disease are required in order to develop new therapeutic strategies. To establish these novel therapeutic approaches animal models of asthma have been developed and refined in the past years [12]. Different animal species have been used so far for these models. Starting with guinea pig models of allergic airway inflammation to assess pharmacological aspects, new models including rats and mice have been developed which mimic major features of allergic asthma. The mouse seems to be the presently preferred species for the investigation of the immunological basis of the disease [12]. However, there are no in-depth bibliometric analysis of the current state of research in this field available. Therefore the present study was carried out to evaluate the role of animal models in the field of asthma research using large scale data analysis and bibliometric approaches including density-equalizing mapping.

## Methods

### Data source

Data was retrieved from the database Web of Science database from the Thomson Institute for Scientific Information (ISI) [13,14].

### Search strategies

For the different searches, phrases joined together with Boolean operators, i.e. AND, OR and NOT using the words “asthma”,” allergic airway inflammation” and “animal model” were used. Also, the species used for experimental studies, such as guinea pigs, rats or mice and other species were used as search terms. In order to approximate the overall number of published items on animal models of asthma, the following phrase was used: “asthma*” OR “allergic airway inflammation”. This search routine was then combined with the following phrase: “animal* model*” OR “ovalbumin” OR “murine* model*” OR “mouse* model*” OR “mice* model*” OR “rat* model*” OR “guinea pig model*” OR “monkey* model*” OR “dog model*”. The asterisk was used to replace the word ending in order to encompass all possible endings (e.g. asthmatic, asthmaticus). Also, the term “ovalbumin” was used in the search strategies since it is the most prominent allergen used in animal models to induce allergy.

In addition, each search was limited to preferred document types using a “refine results” function in order to include only original articles, reviews or abstracts and excluding publication types such as letters, editorials and news reports.

### Time span

The initially analyzed time span included the period from 1900 to 2006. 2007 was not included since the data acquisition is not terminated so far. To examine particular aspects of the retrieved data the time span was partly restricted to a period between 1990 and 2006.

### Citation quantities

Published items were also analyzed using the “citation report” method. This method was used to assess the citations per year of citation, and the average citations per item, indicating the average number of citing articles for all items in the set. It is the sum of the times cited divided by the number of results found.

### Data categorization

All data files were examined concerning a variety of different aspects e.g. the origin (publishing countries), the publication date, the source title, the subject category. Data was transferred to excel charts and visualized in diagrams.

### Density-equalizing mapping

Density-equalizing mapping was used according to a recently published method. In brief, territories were re-sized according to a particular variable, i.e. the number of published items. For the re-sizing procedure the area of each country was scaled in proportion to its total number of published items regarding animal models of asthma. The specific calculations are based on Gastner and Newman's algorithm [15].

## Results

### Total number of published items

**Figure 1:**
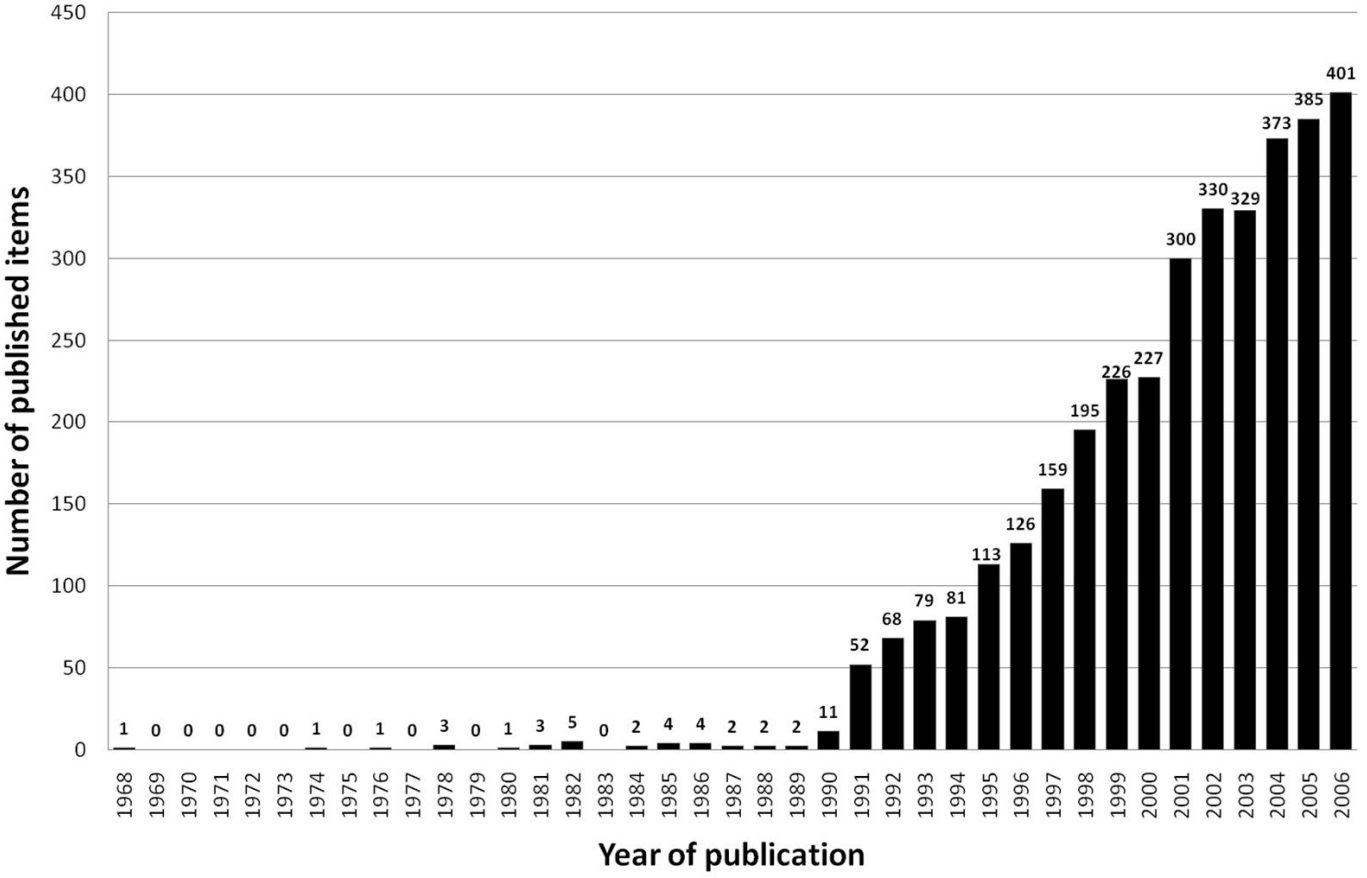
Published items related to animal models of asthma in the Web of Science database 1900-2006.

The number of published items was used as an index of quantity of research productivity and large differences were found: During the period 1900–2006 a number of 78.860 items were published and included in the Web of Science database with the combined words “asthma*” and “allergic airway inflammation” identified in the title, abstract or key words. Within this data file, 3.489 entries were also related to animal models of asthma, as defined by the search routine. The first studies were published in the year 1968 and numbers increased at the beginning of the 1990's (Figure 1).

### Analysis of origin

**Figure 2:**
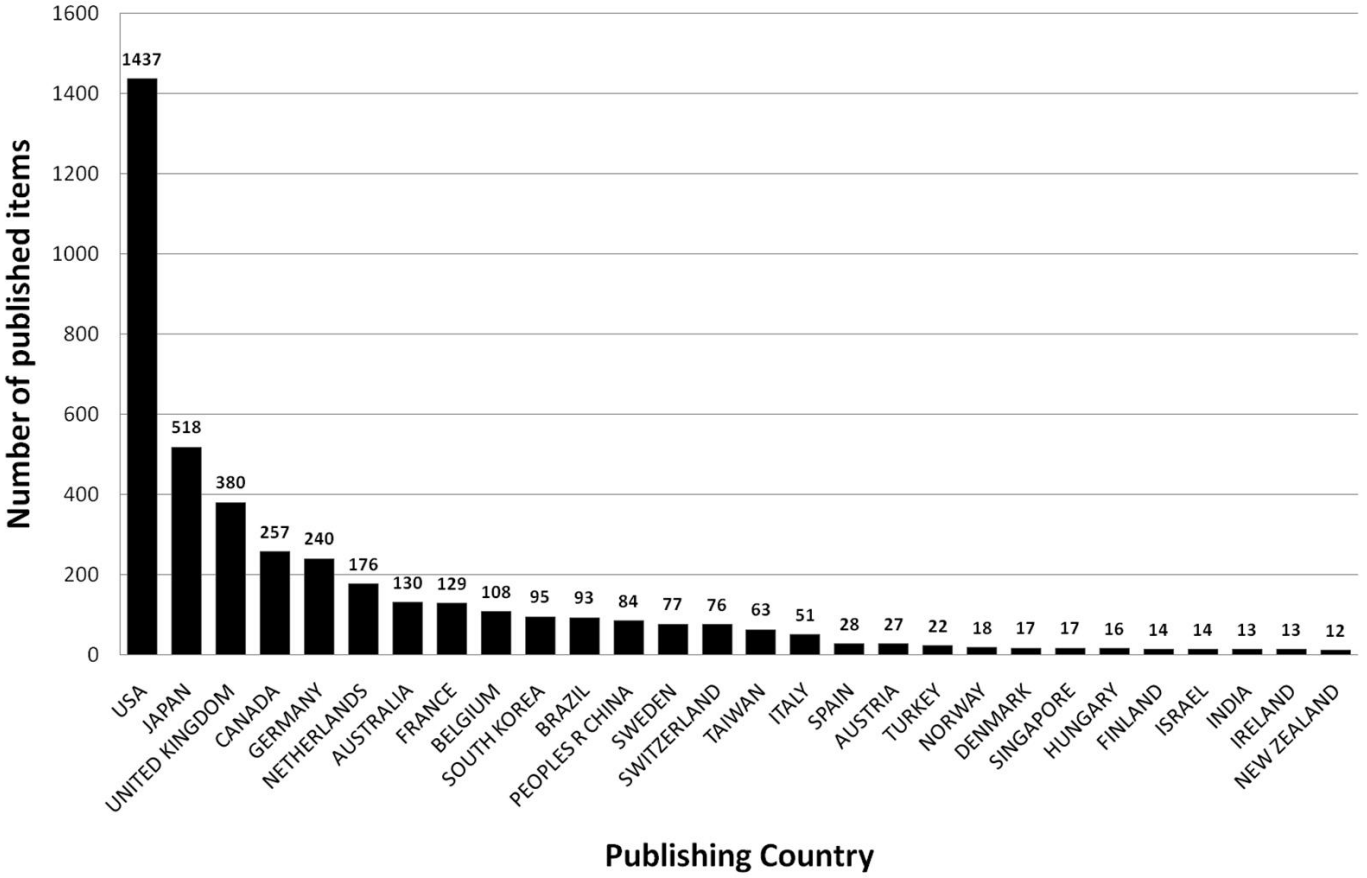
Ranking of country total numbers of published items related to animal models of asthma. Threshold of > 10 published items.

**Figure 3:**
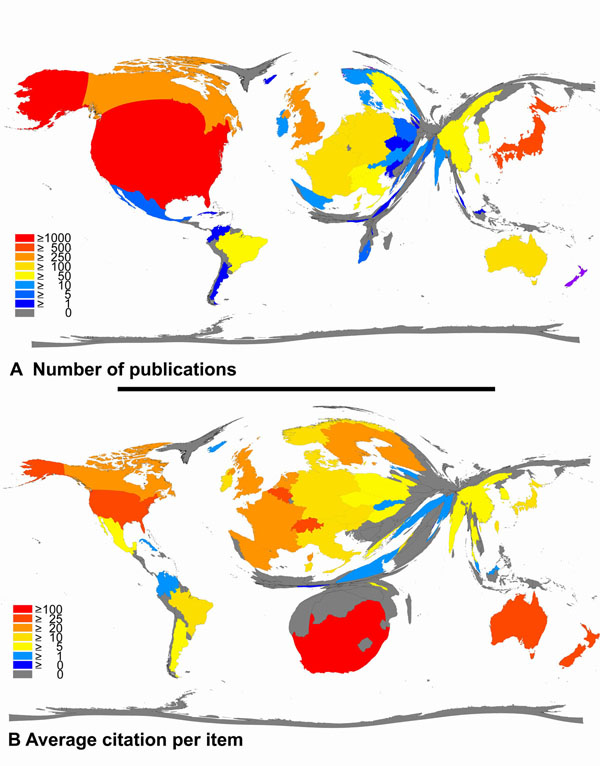
**A.** Density-equalizing map illustrating the number of publications in each particular country. The area of each country was scaled in proportion to its total number of publications regarding animal models of asthma. **B.** Density-equalizing map showing the average citations per item of each particular country. The area of each country was scaled in proportion to its average number of citations per item regarding animal models of asthma.

The 3489 entries originated from 52 countries with the US, Japan and the UK being the most productive countries (Figure 2) participating in 55.8% of all published items. The cumulated publications of the top ten publishing countries encompassed 82.9% of all published items, taking into account that 721 of all filed items were a collaboration of two or more countries. 18 publications could not be assigned to a certain country. Density-equalizing mapping of this set of data demonstrates that a relatively small number of countries is responsible for the majority of research efforts (Figure 3a).

### Citation parameters

The average citation per item was used as an indicator for research quality and differences were found in relation to research quantity figures: When analyzing all 3489 published items regarding the average citation of each item in a country-specific manner, South Africa has the highest average citation rate (132/item) with Switzerland ranking second (30.54/item) and New Zealand ranking third (30.17/item) (Figure 3b). Differences to output quantity (Figure 3a) can be visualized by the use of a density-equalizing calculation to provide a global scheme of the average citations per item of each country (Figure 3b).

**Figure 4:**
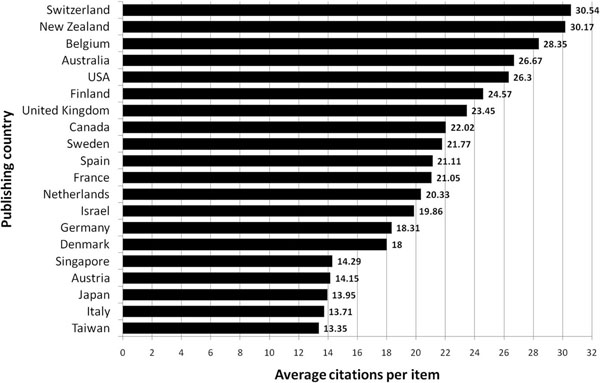
Average citations per item. Threshold excludes countries with < 10 publications.

When a threshold of at least ten publications is introduced, South Africa (8 publications) and Slovenia (1 publication) are not longer ranked in the top ten and Switzerland moves into first position. Additionally Italy and Taiwan enter the list of the 20 countries with the highest ranking (Figure 4).

**Figure 5:**
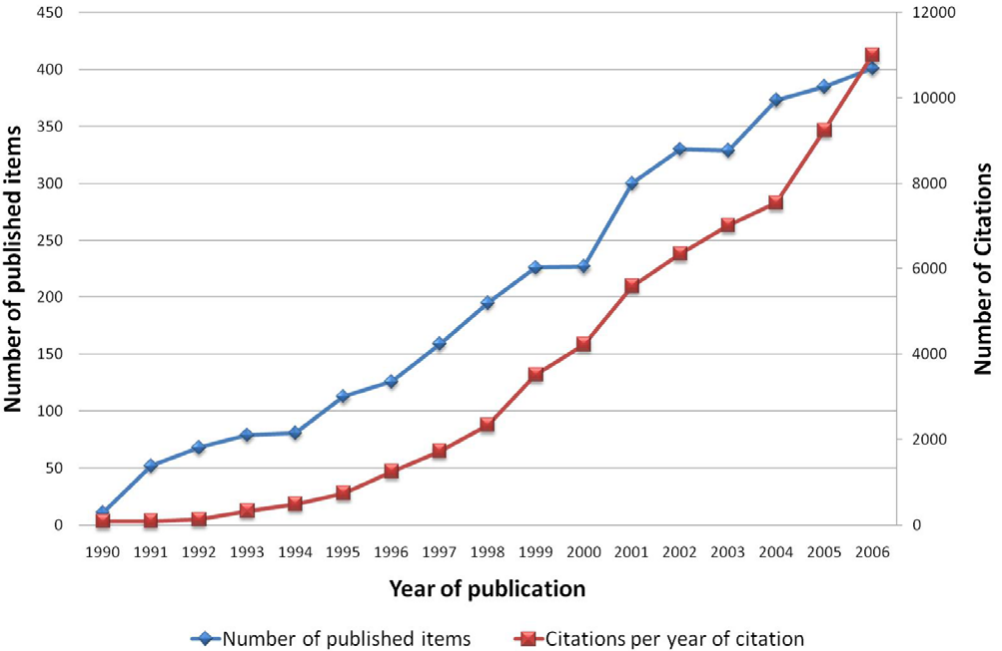
Published items in each year compared to the citations per year of citation (1990-2006)

To assess the reception of the subject matter over the time the citation rates per year were recorded from 1990 to 2006 and a trend of increasing citations was present since 1990 which parallels the increase in published articles (Figure 5).

### Publishing journals

**Figure 6:**
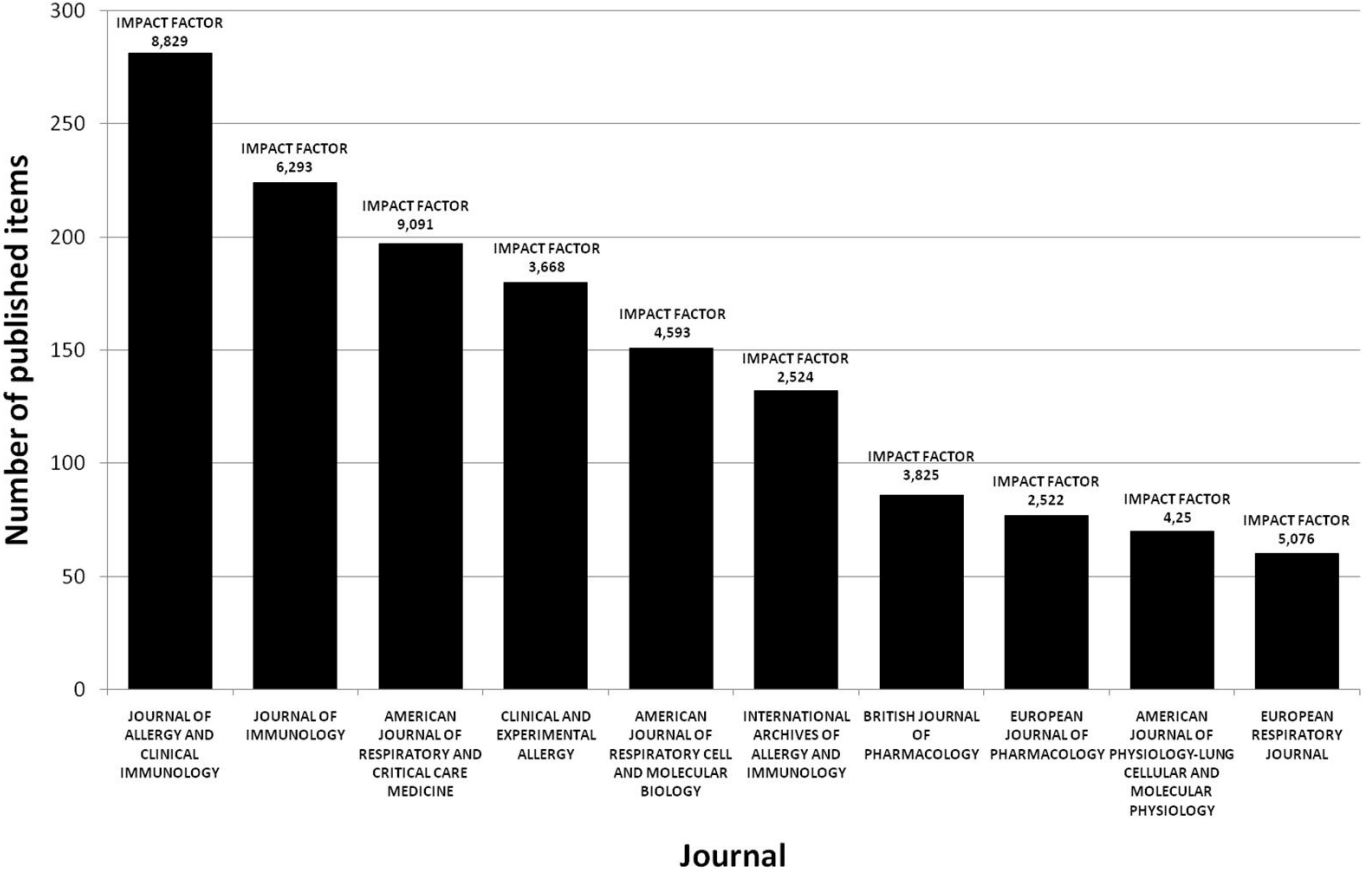
Top ten ranking of sources by the number of published items during the period from 1900 to 2006.

The ten most productive journals include four journals with main focus allergy/immunology, another four with main focus respiratory system and two dealing with pharmacology/pharmacy. Those with main focus on allergy and respiratory system are the leading journals in their subcategory concerning their impact factor in 2006 (9.091 and 8.829) and are well established. The remaining journals with the topics immunology and pharmacology range mid-field in their category with impact factors of 2.522-6.293. (Figure 6).

### Analysis of assigned subject categories

**Figure 7:**
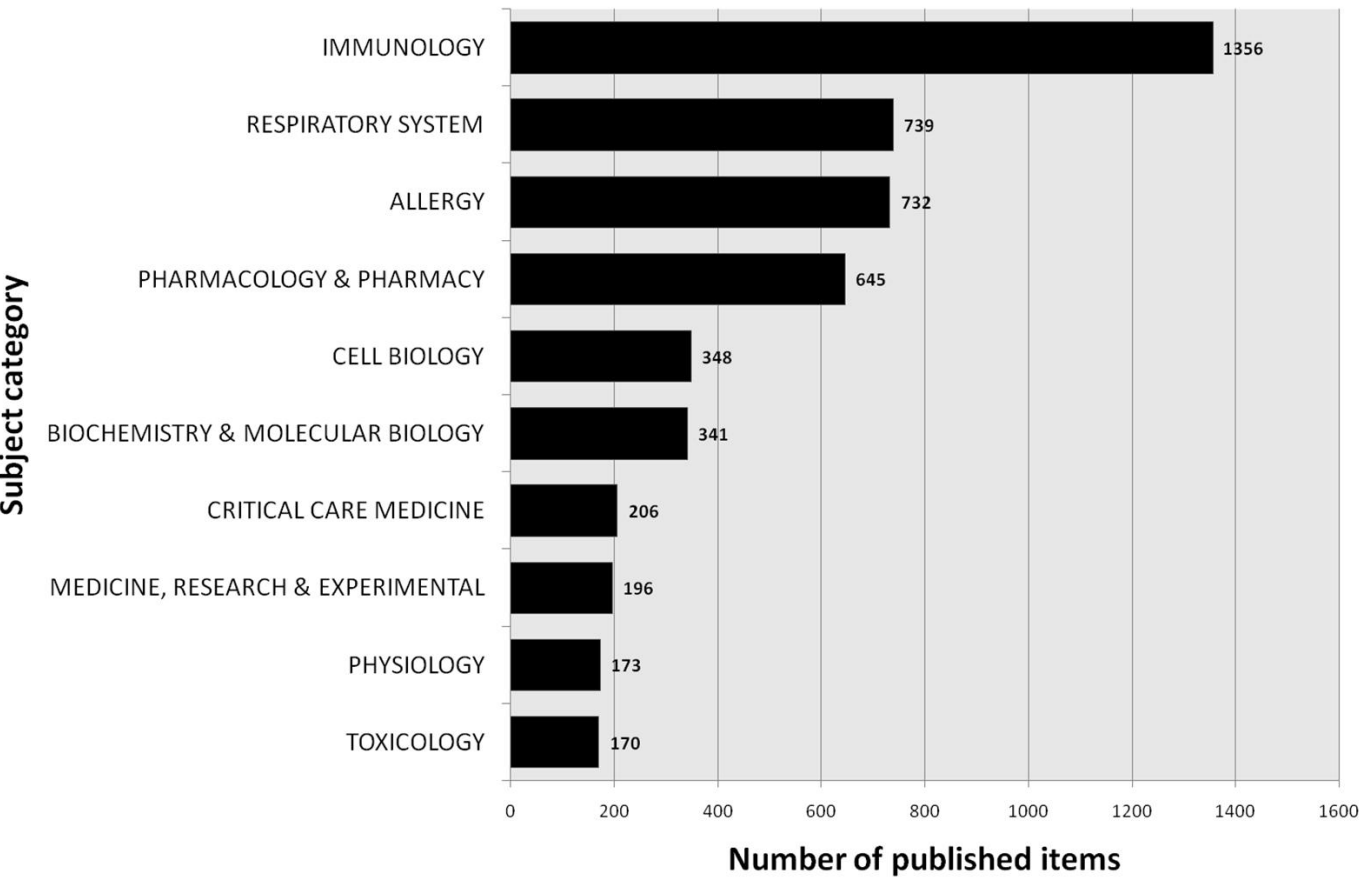
Top ten list of assigned subject categories of published items related to animal models of asthma. Study period from 1900 to 2006.

**Figure 8:**
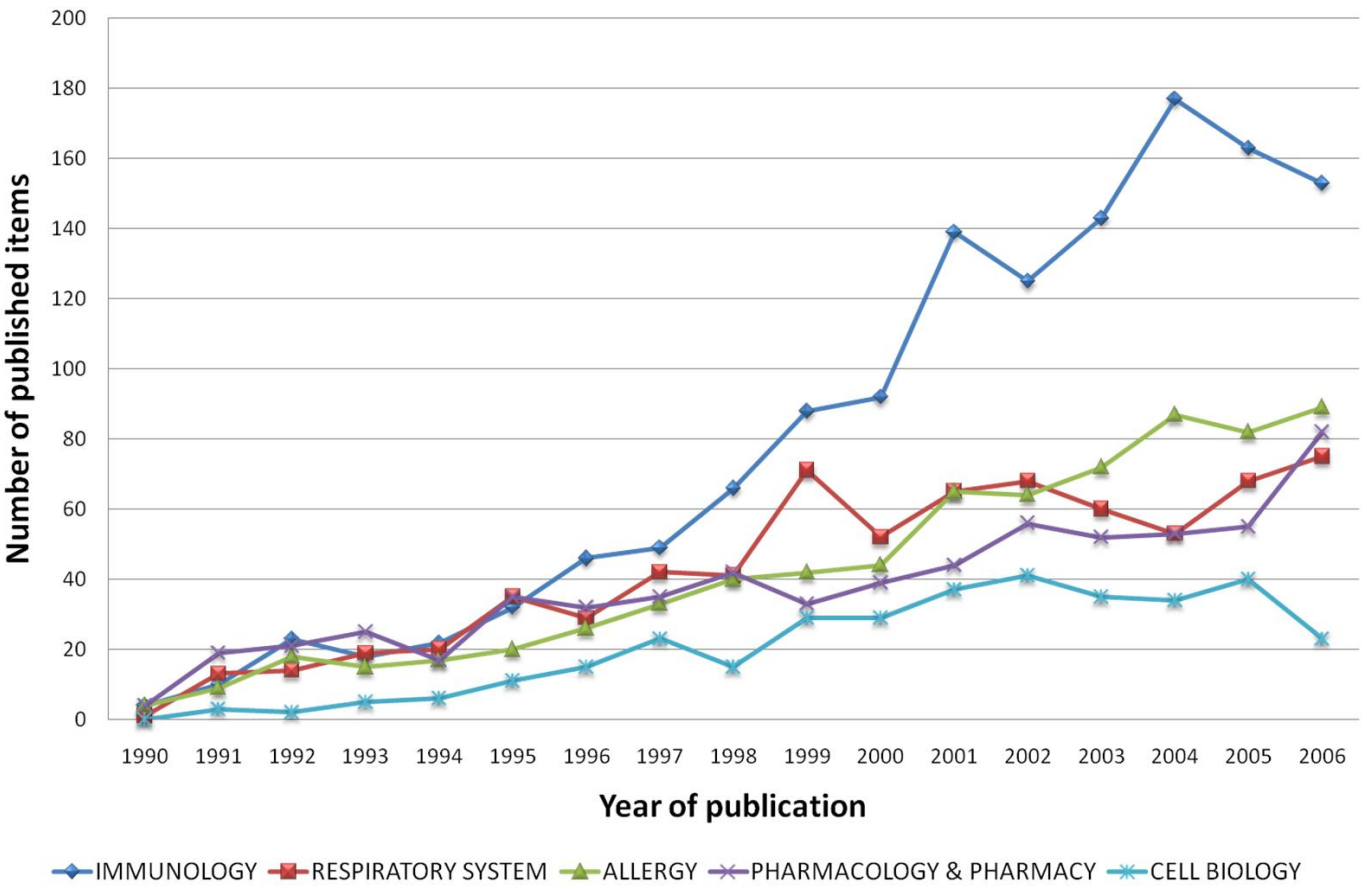
Time dynamics of five selected assigned subject categories. Study period from 1900 to 2006.

In all subject categories examined for published items related to animal models of asthma, immunology ranked first by far, followed by the categories respiratory system, allergy, pharmacology and cell biology (Figure 7). The amount of research with animal models conducted in the field of pharmacology and pharmacy constitutes 18.5% of all analyzed research categories (Table 1). Whereas the number of articles published in the subject category “immunology” increased remarkably since 1997, the subject categories “respiratory system”, “allergy” and “pharmacology/pharmacy” showed a constant but less steep increase. Articles assigned to the subcategory “cell biology” though showed decreasing numbers since 2004 (Figure 8).

**Table 1:** Country-specific relevance of assigned categories in research with animal models. A comparison of five selected countries (table contains multiple nominations).

**Assigned subject category (Top 10)**	**Average percentual amount of all publishing countries**	**Japan**	**United Kingdom**	**Netherlands**	**United States**	**Germany**
**Immunology**	38.9%	43.6%	37.5%	31.3%	38.8%	45.0%
**Respiratory system**	21.2%	18.3%	19.0%	23.2%	23.3%	12.9%
**Allergy**	21.0%	27.2%	19.8%	15.9%	16.6%	30.4%
**Pharmacology and pharmacy**	18.5%	23.6%	29.8%	27.3%	11.3%	13.3%
**Cell biology**	10.0%	8.3%	7.2%	14.2%	12.1%	7.9%
**Biochemistry and molecular biology**	9.8%	8.7%	9.4%	11.9%	11.4%	7.1%
**Critical care medicine**	5.9%	4.8%	3.0%	9.1%	6.8%	4.2%
**Medicine, research and experimental**	5.6%	4.6%	4.0%	1.7%	7.3%	6.7%
**Physiology**	5.0%	1.9%	2.7%	0.6%	7.2%	0%
**Toxicology**	4.9%	4.1%	4.8%	10.2%	5.2%	7.1%

### Species analysis

**Figure 9:**
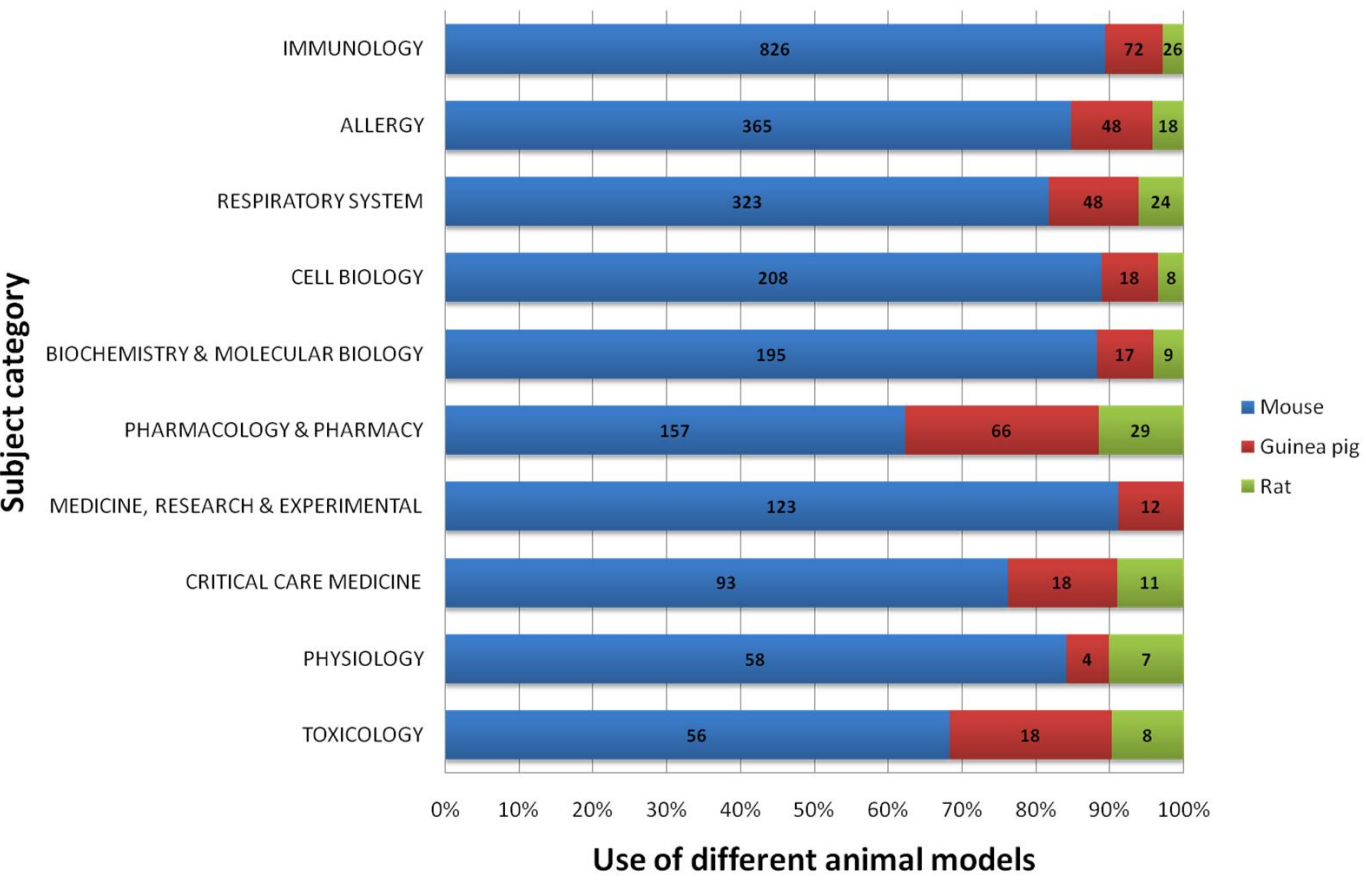
Comparison of the use of different species for animal models of asthma in the ten most common assigned subject categories (in percent and total numbers).

To analyze the role of different laboratory animal species for their use in asthma models, the ten most productive research categories were compared to species. It was found that mice are the overall preferred species, while guinea pigs are mainly used for studies in the field of pharmacy/ pharmacology and toxicology. Rat strains were less relevant (Figure 9).

**Figure 10:**
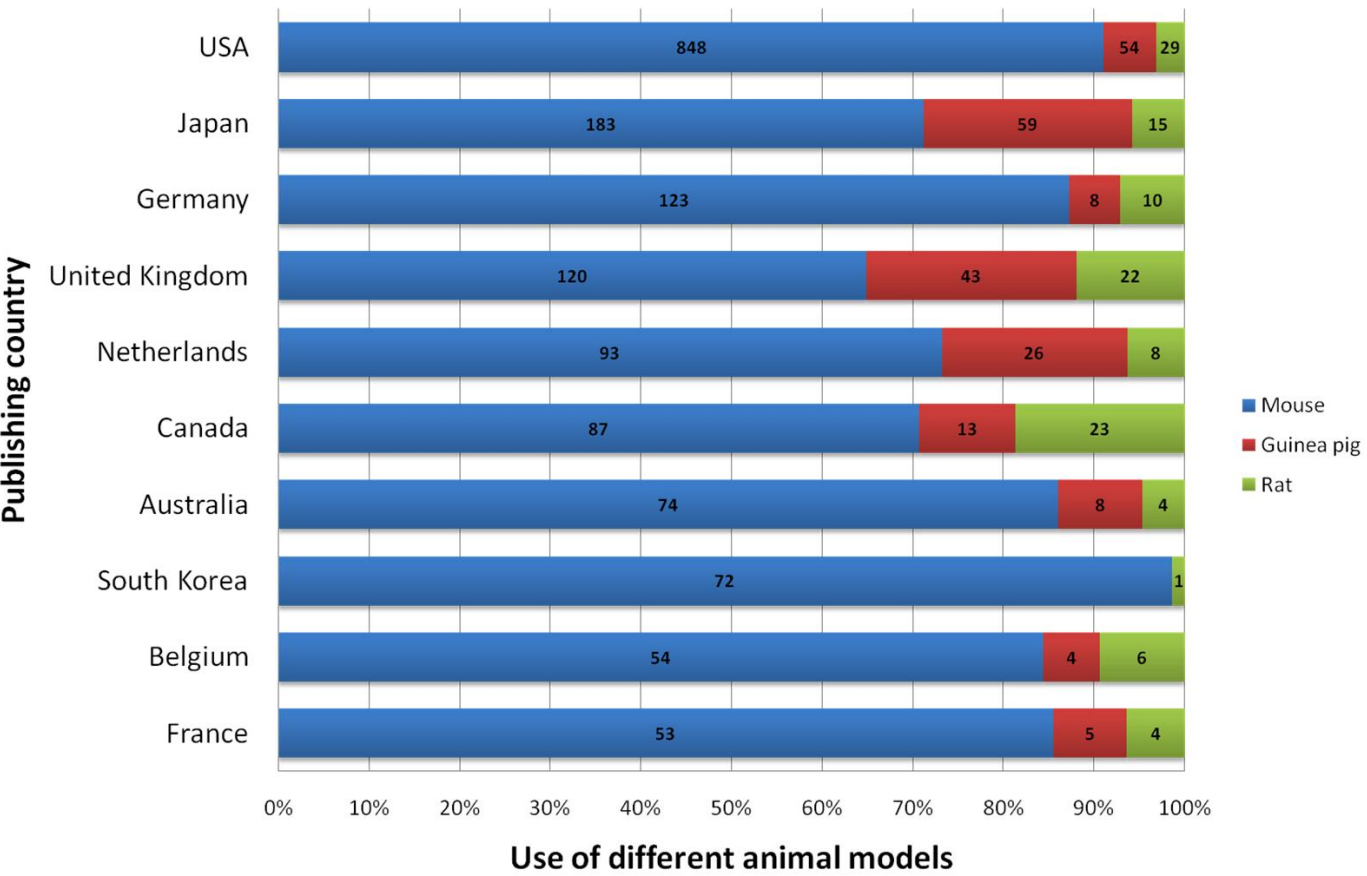
Comparison of the preferred animal model species in the ten most productive countries (in percent and total numbers).

**Figure 11:**
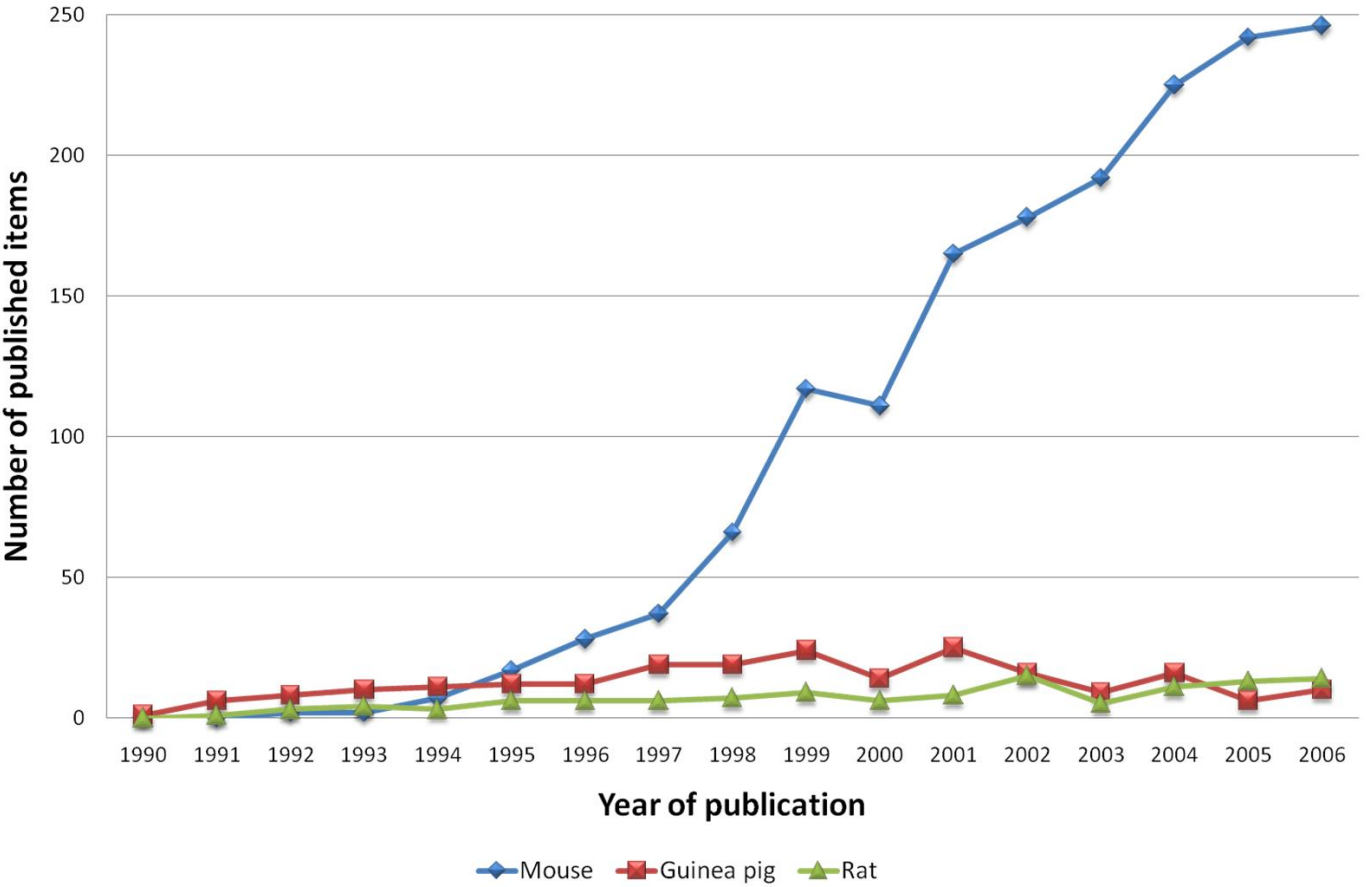
Differences in the development of mice, rats mice and guinea pigs as species for asthma animal models. Study period from 1990 to 2006.

When the use of species was compared to the ten most productive countries it was found that U.S. and German affiliations used mouse models of asthma in more than 85% of their studies, whereas in Japan, the UK and Canada, mouse models were not as dominant (Figure 10). Regarding the use of rats, mice and guinea pigs as animal models of asthma in the period between 1990 and 1994 the importance of all three species seemed to have rather similar priority. With the beginning of 1995, mice started to play a major role and became the most prominent species used in animal models of asthma (Figure 11).

## Discussion

The past decades of research in the field of asthma have been challenged by a number of revolutionary insights into immune mechanisms of the disease. Since this research was mainly performed in animal models using novel tools of molecular biology such as loss-of-function or gain-of-function the number of animal studies using mice as species increased as shown in the present study. The present study provides a precise bibliometric evaluation of the role of animal models in the field of asthma research. So far, this aspect has not been investigated in detail and only reviews have focused on methodological and technical issues of animal models [12,16]. The present methodology is based on internationally established databases such as the Web of Science [13,14] and novel bibliometric tools including density- equalizing mapping [15]. The time span in some search routines was restricted to the period between 1990 and 2006. This was chosen because the worldwide number of published items before 1990 was relatively low.

Generally, there is a constant increase of interest in this field since the beginning of the 1990's. The large interest in the subject can also be seen when the most productive journals are analyzed. Data analysis of productivity parameters shows that research groups from the US maintain a leadership position in research productivity concerning asthma research in general and animal models of asthma in particular, along with the UK. It is notable that Japan ranks fourth in general asthma research (data not shown) and even second when animal models of asthma are focused. The tendency of only a relatively small number of countries contributing the majority of research can also be remarkably illustrated by density-equalizing mapping procedures.

Whereas the number of published items was considered as an index of quantity of research productivity, the average citation per item was used as an indicator for research quality as generally accepted. Therefore all articles were analyzed regarding the average citations of items published in each particular country. Using this average citation per item index without thresholds, South Africa appears to have the highest rank, followed by Switzerland, New Zealand, Belgium and Australia. It has to be annotated, that the results for those countries with a very small amount of published items appear disproportionately high. To objectify these outliers, a threshold of ten published items was introduced and as a result, South Africa (8 publications) and Slovenia (1 publication) are not longer included in the ranking. Switzerland then moves into first position. Additionally, Italy and Taiwan enter the list of the 20 countries with the highest ranking as shown in Figure 4. The leading position of Switzerland seems reasonable when the Swiss institutions are analyzed with internationally renowned institutions such as the Swiss Institute of Asthma Research in Davos (SIAF). Also, Belgium, despite being a relatively small country concerning size and population, houses a number of renowned institutions devoted to asthma research including the University of Ghent or the Catholic University of Leuwen.

When focusing on assigned categories in the database Web of Science related to animal models of asthma, the field of immunology plays a leading role with a steep increase of published articles since 1997. This trend is parallel to the enormous financial input in this field from public and private institutions and to the increasing overall numbers of published studies related to immune mechanisms (data not shown). In contrary, animal model research concerning cell biology seemed to stagnate as illustrated in Figure 8. This result might be biased by an increased focus on immune mechanisms in the field cell biology with an artificial denomination shift from the category cell biology to the category immunology in various studies.

When analyzing the role of different species, it is not surprising that murine models of asthma are the preferred species in all countries and subject categories. This trend is parallel to the increase in studies related to immunology since the mouse is the best species to generate gene-depleted strains. However, guinea pigs are still often chosen as asthma model species by countries such as Japan, the UK and the Netherlands as shown in Table 1. This is most probably due to the fact that a major interest of asthma research in these three countries is the area pharmacology. I.e. results for the UK show that the field of pharmacology constitutes 29.8% of overall research. Similar numbers can be found for the Netherlands (27.3%) and Japan (23.6%) The reason for this strong interest can also be attributed to single institutions in these countries. I.e. the Dutch University of Utrecht harbors an internationally renowned department of pharmacology with the focus on airway pharmacology. Pharmacology is also a focus of established institutions in the UK such as the National Heart and Lung Institute in London. Thus, pharmacology as an area of research related to animal models of asthma ranks fourth when all publications and countries are assessed, but second in the UK or the Netherlands. Strikingly, rat models seem to have a noteworthy impact on research only in Canada as illustrated in Figure 10. US or Germany – these are countries with a predominant use of mouse models and only a minor use of guinea pig models — also show lesser interest in the field of pharmacy and pharmacology (11.3% and 13.3%, respectively) when compared to the UK, the Netherlands or Japan as illustrated in Table 1.

The presently discovered enormous increase in studies using murine models of asthma is definitely related to the increase in immunological studies of the disease. However, most novel immunomodulatory drug classes for asthma therapy failed to reach clinical practice [17]. It may therefore be asked if the global research efforts that tried to identify novel single immune targets were too reductionistic. It needs to be stated in this respect that the current rate of introduction of novel compounds to the pharmaceutical market is lower than at any time in the past 50 years [17] although the overall number of new discoveries concerning immune mechanisms and murine animal studies raises. Within a complex disease such as asthma not only inflammatory cells but also other systems might play a crucial role. Since mice do not cough and also do not have glandular structure as humans, the future research should reappraise other species. In this respect, the guinea pig offers a greater proximity to the human situation since they i.e. share a similar airway innervation. They also have common symptoms of asthma such as cough which mice do not have.

## Conclusion

The present study represents the first detailed bibliometric analysis of the role and impact of animal models of asthma. The data shows a strong increase in research productivity. Using science citation analysis it can be assumed that there is an increase in the interest in results of animal studies. While the majority of data originates from the US, smaller countries such as Switzerland take a lead in citation per item rankings.

## Competing interests

The authors declare that they have no competing interests.

## Authors' contributions

BGK, CS and TCF and designed the study. JAB, NN, CS and BGK performed the search routines and constructed the different data files. CP performed pilot data search routines and analysis.
